# The Association between Drought Exposure and Respiratory-Related Mortality in the United States from 2000 to 2018

**DOI:** 10.3390/ijerph20126076

**Published:** 2023-06-07

**Authors:** Yeongjin Gwon, Yuanyuan Ji, Jesse E. Bell, Azar M. Abadi, Jesse D. Berman, Austin Rau, Ronald D. Leeper, Jared Rennie

**Affiliations:** 1 Department of Biostatistics, College of Public Health, University of Nebraska Medical Center, Omaha, NE 68198, USA; yuanyuan.ji@unmc.edu; 2 Daugherty Water for Food Global Institute, University of Nebraska, Lincoln, NE 68588, USA; 3 School of Natural Resources, University of Nebraska, Lincoln, NE 68588, USA; 4 Department of Environmental Agriculture Occupational and Health, College of Public Health, University of Nebraska Medical Center, Omaha, NE 68198, USA; 5 Environmental Health Sciences, School of Public Health, University of Alabama at Birmingham, Birmingham, AL 35233, USA; aabadi@uab.edu; 6 Division of Environmental Health Sciences, School of Public Health, University of Minnesota, Minneapolis, MN 55455, USA; berma186@umn.edu (J.D.B.); rauxx087@umn.edu (A.R.); 7 North Carolina Institute for Climate Studies, North Carolina State University, Raleigh, NC 27695, USA; ronnieleeper@cicsnc.org; 8 NOAA’s National Centers for Environmental Information, Asheville, NC 28801, USA; jared.rennie@noaa.gov

**Keywords:** respiratory mortality, risk ratio, USDM, EDDI, climate region, drought

## Abstract

Climate change has brought increasing attention to the assessment of health risks associated with climate and extreme events. Drought is a complex climate phenomenon that has been increasing in frequency and severity both locally and globally due to climate change. However, the health risks of drought are often overlooked, especially in places such as the United States, as the pathways to health impacts are complex and indirect. This study aims to conduct a comprehensive assessment of the effects of monthly drought exposure on respiratory mortality for NOAA climate regions in the United States from 2000 to 2018. A two-stage model was applied to estimate the location-specific and overall effects of respiratory risk associated with two different drought indices over two timescales (the US Drought Monitor and the 6-month and 12-month Evaporative Demand Drought Index). During moderate and severe drought exposure, respiratory mortality risk ratio in the general population increased up to 6.0% (95% Cr: 4.8 to 7.2) in the Northeast, 9.0% (95% Cr: 4.9 to 13.3) in the Northern Rockies and Plains, 5.2% (95% Cr: 3.9 to 6.5) in the Ohio Valley, 3.5% (95% Cr: 1.9 to 5.0) in the Southeast, and 15.9% (95% Cr: 10.8 to 20.4) in the Upper Midwest. Our results showed that age, ethnicity, sex (both male and female), and urbanicity (both metro and non-metro) resulted in more affected population subgroups in certain climate regions. The magnitude and direction of respiratory risk ratio differed across NOAA climate regions. These results demonstrate a need for policymakers and communities to develop more effective strategies to mitigate the effects of drought across regions.

## 1. Introduction

Over the past 30 years, there has been a significant increase in deaths and disability caused by chronic respiratory diseases globally [1]. Studies have identified environmental exposure, including pollution, as one of the major risk factors that can escalate respiratory disease. Chronic respiratory diseases are a significant public health issue, with an estimated 3.9 million deaths worldwide in 2017, representing 7% of all deaths worldwide [1].

The interest in quantifying health risks associated with climate-related exposures has grown as the impacts of climate change become more apparent. Identifying and understanding the health outcomes from climate exposures are important for implementing mitigation strategies for at-risk populations in order to reduce future extreme weather-related risks. Numerous studies and assessments have tried to assess the health effects of climate-related exposures, including heat, extreme precipitation, and air quality [2,3,4,5,6,7,8,9,10,11,12]. We refer here to two recent review articles that have assessed the risk to human health posed by extreme weather and climate exposures with regard to their public health implications [5,13]. However, there is limited research investigating the link between drought exposure and health outcomes, especially in terms of respiratory mortality [14,15,16]. This lack of research is likely a combination of the complexity of the pathways to health outcomes from drought and the lack of attention from public health officials towards drought.

Drought is a unique climatic hazard with strong spatial and temporal variability across different regions. However, there is little guidance on the selection and application of drought indices for health risk assessment [17]. In recent years, a significant number of epidemiological studies have examined the potential relationship between drought exposure and mortality both in the U.S. and globally. Berman et al. (2017) found that mortality risk increased during worsening drought periods in areas of the western United States with less exposure to drought [16]. Lynch et al. (2020) identified that drought periods had a statistically significant association with all-cause mortality in the white population aged 25–64 in the United States [18]. Salvador et al. (2019, 2020) showed that longer-term drought and extended drought periods increased daily mortality risk for inland provinces and regions in Spain for populations aged 65 years and older [19,20]. More recently, the risk related to all-cause mortality, non-external circulatory, and respiratory mortality were assessed due to drought exposure in Portugal and Brazil [21,22]. The authors found that older populations aged 65 years or over had a significantly higher respiratory mortality risk under drought conditions. Abadi et al. (2022) did not find a statistically significant association between drought and all-cause mortality in the total population of Nebraska in the United States [14]. However, long-term drought was associated with a higher all-cause mortality rate in white individuals aged 25–34 in metro countries and aged 45–54 in non-metro counties. In spite of these studies, we are aware that there remains a substantial gap in evaluating the specific health outcomes associated with drought, especially for different regions of the United States.

Drought can have severe impacts on human health, particularly on respiratory health. As water becomes scarce during a drought, levels of air pollution can increase due to dust, smoke, and other particulate matter [23,24]. The presence of these pollutants in the air can trigger respiratory issues such as asthma and bronchitis or aggravate existing respiratory conditions [24,25]. Additionally, droughts can lead to the proliferation of airborne allergens such as pollen, further exacerbating respiratory issues. Moreover, drought-induced wildfires, which are becoming more common with climate change, can release harmful gases and particulate matter that can harm the respiratory system [5]. In summary, droughts have significant impacts on respiratory health, and it is essential to understand and address these impacts in order to protect public health.

In this article, we investigated the effects of drought exposure on respiratory mortality from 2000–2018 in the National Ocean and Atmospheric Administration (NOAA) climate regions in the U.S. Our investigation focused on these health risks using different drought indicators, namely, the United States Drought Monitor (USDM) and the 6-month and 12-month Evaporative Demand Drought Index (EDDI), to capture the severity of medium- to long-term droughts. To the best of our knowledge, this is the first climate region-level study to evaluate the health risks associated with drought exposure.

## 2. Materials and Methods

### 2.1. Health Data and Study Population

In this retrospective study, mortality data were extracted from the National Center for Health Statistics (NCHS) from January 2000 to December 2018. We generated monthly county-level mortality counts for respiratory disease (ICD-10 codes J09–J98) according to the International Classification of Diseases, 10th revision (ICD-10 codes) in the United States. We then stratified the mortality counts based on four age groups (0–19, 20–39, 40–64, and 65+ years), three race groups (White, Black, and Other) and sex (Male and Female). No additional demographic or health variables were available. County-level annual population estimates obtained from the surveillance, epidemiology, and end results (SEER) program were aggregated for each population subgroup based on age, sex, and race. We used year-specific population estimates for all the months in each year. The NCHS 2013 binary urban/rural classification codes were used to classify counties into metropolitan and non-metropolitan [26]. Metropolitan counties were divided into four categories: large central metro, large fringe metro, medium metro, and small metro based on population size. Non-metropolitan counties were further differentiated into the two subcategories of micropolitan or noncore with population less than 50,000.

### 2.2. Environmental Exposure

Although no gold standard exists for assessing drought indices, the USDM has been widely used due to its robustness compared with other drought metrics [27,28]. The USDM is a collaborative effort between the National Oceanic Atmospheric Administration (NOAA), the U.S. Department of Agriculture (USDA), and the National Drought Mitigation Center (NDMC) that has been providing weekly updates of drought conditions since 2000 [29]. Using a convergence of evidence approach, the USDM blends moisture deficits from across the hydrological cycle (i.e., precipitation, soil moisture, evaporation, etc.) with drought reports from local experts to categorize drought conditions into one of six categories: wet to normal conditions (None), abnormally dry (D0), moderate (D1), severe (D2), extreme (D3), and exceptional (D4). We reclassified these USDM measures into monthly binary and three-level categories for this assessment. A binary measure was estimated based on the frequency of the drought status within a given month and county. ‘No drought’ was defined if the the no drought and D0 conditions were more frequent for a particular week within a given month and county than all D1 to D4 conditions. Otherwise, that week’s the status was labeled as being in a drought condition. The three-level categorical status was determined as follows: (i) no drought according to the binary measure; (ii) moderate drought (with binary drought condition and the sum of frequencies of D1 and D2 greater than that of D3 and D4); and (iii) severe drought (with binary drought condition and the sum of frequencies of D3 and D4 greater than that of D1 and D2).

The EDDI measures drought signals by assessing how atmospheric evaporative demand (E0) responds to surface drying anomalies [30,31]. This index provides near-real-time information for the entire U.S., and is available in various timescales from weekly through monthly. Short-term EDDI, for example, indicates the atmospheric conditions that can lead to flash droughts, while longer-term EDDI may indicate the development of more sustained drought conditions. Although EDDI is a continuous value, it has categories mirroring the same thresholds in USDM that are determined based on the distribution of aggregated evaporative demand values [30,31]. This supports equivalent comparison between USDM and EDDI drought conditions.

**Figure 1:**
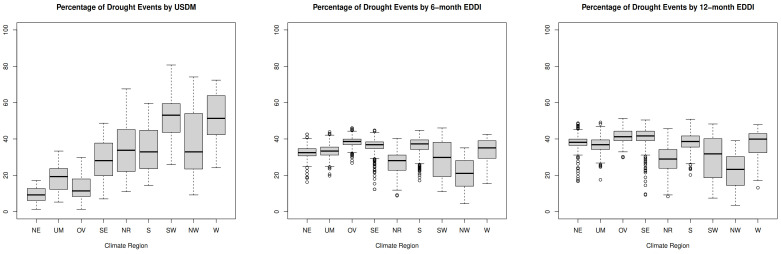
The percentage of months exposed to binary drought events as described using the three drought indicators and stratified by NOAA climate region (2000–2018). Abbreviations indicate climate regions (NE: Northeast, UM: Upper Midwest, OV: Ohio Valley, SE: Southeast, NR: Northern Rockies and Plains, S: South, SW: Southwest, NW: Northwest, W: West) and drought indices (USDM = US Drought Monitor, EDDI = Evaporative Drought Demand Index).

The USDM combines different drought metrics across the hydrological cycle, whereas EDDI focuses solely on evaporation. This leads to distinct spatial patterns in drought, as shown in Figure 1, and motivates research into how these differences affect the assessment of health risks. In addition, the monthly mean temperature anomaly from the NOAA’s Nclimgrid product at 5 km grid cell resolution was included [32]. We then calculated a monthly county-level temperature anomaly metric using the zonal averages of all grid cells falling within a county boundary.

### 2.3. Statistical Modeling

We used a two-stage model to estimate county-level and overall effects between different levels of drought exposure and respiratory mortality risk. In the first stage, we estimated location-specific associations between drought exposure and respiratory mortality in each county using a quasi-Poisson regression model. We included a natural cubic spline of the month with three degrees of freedom to control the long-term time trend, the second degree polynomial of temperature anomalies, and indicator variables to control for calendar year effect. The drought exposure level (moderate and severe drought) was used as the categorical variable. The value of the logarithm for population size in each county was used as an offset variable.

In the second stage, we applied a meta-analysis approach to the county-specific estimates to calculate the overall estimates and NOAA climate region-specific estimates. In particular, we combined county-specific effect estimates using a Bayesian linear regression model and used random effects to estimate the overall effects (i.e., the overall risk ratio). Because this model formulation could lead to the county-specific estimates being interpreted as the county-specific risk ratio at log-scale, we considered the impacts at an exponential to compute the overall risk ratio. The variability of the random effects was used to quantify the heterogeneity between counties within each climate region. We additionally stratified the populations by age group, race, sex, and urbanicity. In addition, it needs to be emphasized that we performed a sensitivity analysis to assess the overall risk ratio while adjusting for high monthly temperature events. To identify the status of a temperature event, we created a new indicator variable based on the 90th-percentile monthly mean anomaly temperature. This variable plays the concurrent role to that of drought exposure, capturing the effect of high temperatures.

Finally, we would remind readers that the posterior distribution is the main inference tool of the Bayesian method, and that the key quantities for interpretation are referred to differently. Posterior estimates and the 95% credible interval (Cr) in Bayesian analysis are analogous to estimates and a 95% confidence interval (CI) in classical statistics. Statistical significance is determined if the corresponding 95% Cr of the estimates do not include the value of zero. The R statistical software program (version 4.2.2) was used to generate all figures. For all statistical analyses, we used Statistical Analysis Software (SAS version 14.2) with the PROC GLIMMIX and PROC MCMC procedures.

## 3. Results

### 3.1. Data Cleaning and Descriptive Statistics

Table 1 provides summary statistics for respiratory death counts in the study population during the 2000–2018 study period. The total number of respiratory deaths in the U.S. during this period was 4,662,776. Overall, the observed and mean respiratory death counts showed a marked increase in respiratory mortality among the older population, with individuals ages 40 to 64 and over 65 having higher mortality rates. In terms of ethnicity, mortality rates were higher for the White population group than for the Blacks or Other groups. Among the age groups, the youngest group (0–19 years) had the lowest percentage of respiratory mortality events, at 5.7%, while the group over 65 years of age had the highest percentage at 81.85%. Approximately 90.25% of mortality risk was in the white population, while only 1.96% and 7.79% of risk was represented by the Black and Other populations, respectively. Both female and male populations showed similar respiratory mortality rates at 52% and 48%, respectively (see Table 1).

Table 2 presents the frequency of total drought exposure by NOAA climate regions. Overall, the 12-month EDDI has more drought months than the USDM and 6-month EDDI, as indicated by its lower frequency of no-drought events. The EDDI uses atmospheric variables such as temperature, humidity, wind speed, and solar radiation to estimate the evaporative demand, or the amount of water that is being demanded by the atmosphere. Therefore, EDDI can provide an early warning of drought conditions before they are fully manifested in other drought indicators such as the USDM. For this reason, the EDDI shows a higher percentage of drought events compared to the USDM. Moreover, the monthly level of drought events summarized by drought exposure metrics shows a distinct spatial pattern in the study area. Stratification by NOAA climate region reveals that Northeast, Ohio Valley, South, Southeast, and Upper Midwest have a higher level of monthly total drought events (moderate and severe) compared to other regions based on the 12-month EDDI measure. This reveals that EDDI defines these climate regions as being more prone to drought exposure than others, which differs from the USDM.

Figure 1 displays the distribution of percentage area experiencing binary drought events during different drought exposures by NOAA climate regions. The percentage was calculated based on the total number of drought events experiencing either moderate or severe drought in the counties over the entire study period, summarized by NOAA climate region level. The percentage presented in the map is provided in Figure S1 of Supplementary Materials. Various spatial patterns of three different drought types were identified across climate regions (Figure 1 and Figure S1). The Northeast, Upper Midwest, Ohio Valley, and Southeast experienced more drought events on the 6-month and 12-month EDDI compared to the USDM, while the USDM captured more drought events in the Southwest, Northwest, and West. However, the Northern Rockies and Plains had similar percentages of drought events in all three drought indexes.

### 3.2. Association between Drought Exposure and Respiratory Outcomes

For the second-stage model fitting, we applied a different screening criterion to determine the counties to be included in the analysis. Rather than using strict inclusion criterion based on the population size of the counties (e.g., including counties with populations greater than 12,500, 25,000, or 100,000), we included those counties for which estimate of drought exposure was in the −1.5 to 1.0 range and the standard error was less than 1.0 on a logarithm scale. Our analysis found that 2290, 1635, and 597 counties were included in the first-stage model after applying the above population size thresholds, which disregarded more than 30% of the counties. Table S1 in the Supplementary Materials represents the number of counties included in the analysis. Based on our findings, this approach maintained most location-specific estimates (over 90%, more than 2800 county estimates) from the first-stage model, resulting in improved statistical power for the second-stage analysis.

**Figure 2:**
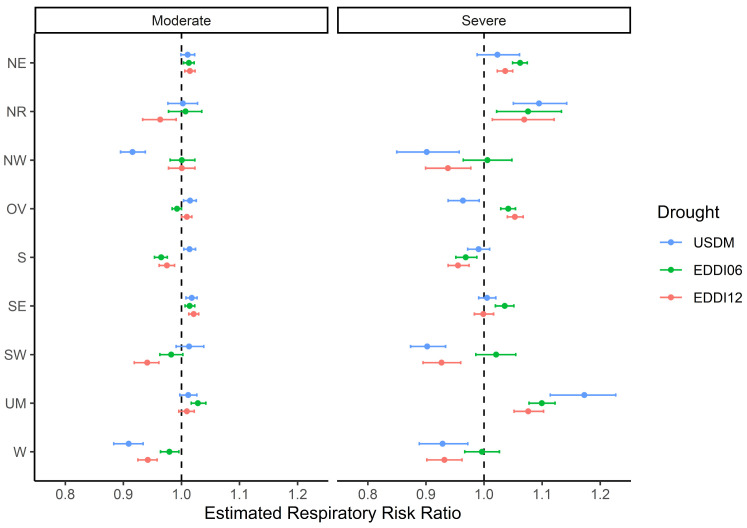
Overall effects of the respiratory mortality risk ratios and their corresponding 95% credible intervals by NOAA climate region and three drought exposure measures. The color of the risk ratio indicates the relationship with different drought indicators (blue = United States Drought Monitor, green = 6-month EDDI, red = 12-month EDDI).

Figure 2 shows the posterior estimates and corresponding 95% Credible (Cr) intervals of the estimated overall effects of the three drought exposure levels by NOAA climate region. The estimates are reported in Table S2 of the Supplementary Materials. Statistical significance was determined based on whether the corresponding 95% Cr of the estimated risk ratios did or did not include the value of zero. Results from the second-stage model show that the overall risk ratios differ in magnitude and direction by NOAA climate region. In Figure 2, it can be seen that four climate regions (Northern Rockies and Plains, Upper Midwest, Northeast, and Ohio Valley) had positive respiratory mortality associated with higher-intensity droughts conditions. The Northern Rockies and Plains and the Upper Midwest had increased respiratory mortality risk ratio for all three severe drought exposures. Both regions had a significantly increased risk ratio of 9.0% (95% Cr 4.9 to 13.3), 7.3% (95% Cr 2.1 to 12.5), and 6.7% (1.4 to 11.4) in the Northern Rockies and Plains and of 15.9% (95% Cr 10.8 to 20.4), 9.5% (95% Cr 7.5 to 11.5), and 7.3% (95% Cr 5.0 to 9.8) in the Upper Midwest with the three indicators of severe drought (Figure 2 and Table 2). Moreover, the risk ratio was significantly elevated to 6.0% (95% Cr 4.8 to 7.2) and 3.6% (95% Cr 2.2 to 4.8) in the Northeast and 4.1% (95% Cr 2.8 to 5.3) and 5.2% (95% Cr 3.9 to 6.5) in the Ohio Valley with severe 6-month EDDI and 12-month EDDI. The Southeast had an increased risk ratio by 3.5% (95% Cr 1.5 to 5.0) from severe 6-month EDDI drought. However, severe drought exposure significantly decreased the risk ratio by drought type in the Northwest, Ohio Valley, Southwest, and West (Figure 2 and Table 2). Although severe droughts had adverse effects on respiratory mortality in certain climate regions, moderate drought increased the risk ratio across climate regions as well. The Northeast and Southeast saw increased risk ratio when experiencing moderate drought by up to 1.8%, 1.4%, and 2.1%, respectively (Figure 2 and Table 2). These results indicate that the respiratory risk ratio varies with drought exposure in different geographical regions.

### 3.3. Stratification Analysis by Age Group, Race, Sex, and Urbanicity

**Figure 3:**
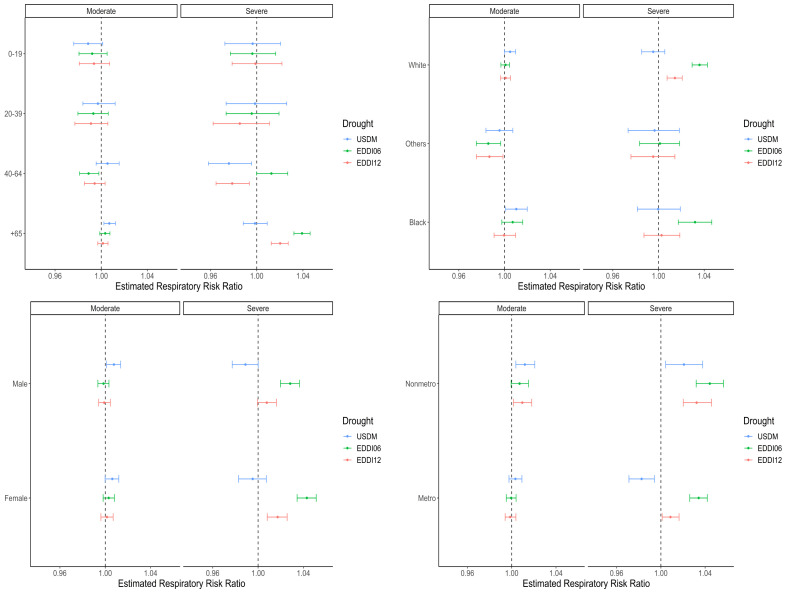
Posterior estimates of the risk ratio associated with different drought exposures for respiratory mortality stratified by age group, sex, urbanicity, and race.

We performed stratification analysis to evaluate the effects of drought exposure on respiratory risk by subgroup. Figure 3 exhibits the overall posterior estimates and corresponding 95% credible intervals stratified by age group, race, sex, and urbanicity. The results, including estimated incidence risk ratios and 95% credible intervals for these analyses, can be found in Tables S3–S6 of the Supplementary Materials.

The results by age group showed that ages 0–19 and 20–39 had decreased respiratory mortality risk ratio from moderate drought and the same direction of the risk ratio for both moderate and severe drought; however, these associations were not statistically significant. On the other hand, the respiratory mortality risk ratios by drought intensity exposure were statistically significant in the opposite direction for ages 40–64. Populations over 65 years of age had increased risk ratios of 3.8% (95% Cr 3.2 to 4.5) and 2.0% (95% Cr 1.3 to 2.7) from higher-intensity EDDIs (Figure 3 and Table S2). A similar positive association was identified during USDM (D1 and D2) drought, with the risk ratio increased by 0.7% (95% Cr 0.2 to 1.2).

The White population subgroup showed a statistically significant increase in respiratory mortality risk of 0.5% (0 to 1.0) with moderate USDM drought and 3.5% (95% Cr 2.9 to 4.2) and 1.4% (95% Cr 0.7 to 2.1), respectively, with both types of severe EDDI drought (Figure 3 and Table S4). The Black population showed greater respiratory risk of 1.0% (95% Cr 0.1 to 2.0) and 3.2% (95% Cr 1.7 to 4.6) with moderate USDM and severe 6-month EDDI drought. However, the Other population subgroup showed a decreased risk of 1.4% (95% Cr −2.5 to −0.3) and 1.3% (95% Cr −2.5 to −0.1) from moderate 6-month EDDI and 12-month EDDI drought.

Statistically significant positive respiratory mortality associations were identified in both the male and female population subgroups with moderate USDM and severe EDDI drought. The estimated respiratory risk increased by 0.8% (95% Cr 0.1 to 1.4) for male and 0.6% (95% Cr 0 to 1.2) for female with moderate USDM drought (Figure 3 and Table S5). However, for severe USDM drought, the male population showed a decreased risk ratio of 1.1% (95% Cr −2.3 to 0). Male and female populations both shows increased risk ratio of 2.8% (95% Cr 2.0 to 3.6) and 4.2% (95% Cr 3.4 to 5.0), respectively, during severe 6-month EDDI drought and 0.8% (95% Cr 0 to 1.6) and 1.7% (95% Cr 0.8 to 2.5), respectively, during severe 12-month EDDI drought. Although the direction of the respiratory mortality risk ratio for male and female differed for moderate 6-month EDDI drought, these associations were not statistically significant.

While the respiratory risk between metro and non-metro counties differed by intensity of drought exposure, most of the drought exposure effects were statistically significant. Both types of severe EDDI drought significantly increased the estimated risk ratio, by 3.4% (95% Cr 2.6 to 4.1) and 0.9% (95% Cr 0.1 to 1.6), respectively, in metro and 4.3% (95% Cr 3.1 to 5.5) and 3.2% (95% Cr 2.0 to 4.5), respectively, in non-metro counties (Figure 3 and Table S5). USDM severe drought increased the risk ratio by 2.1% (95% Cr 0.4 to 3.7) in non-metro counties, while it decreased the risk ratio by 1.8% (95% Cr −2.9 to −0.6) in metro counties. USDM moderate drought was found to significantly escalate risk in non-metro counties by 1.2% (95% Cr 0.3 to 1.9). Although metro counties saw a risk ratio increase of 0.3% from moderate USDM drought, this was not statistically significant.

## 4. Summary and Discussion

In this article, we examined changes in respiratory mortality associated with drought exposure across all NOAA climate regions in the United States from 2000 to 2018. Our assessment is a comprehensive evaluation of a large-scale geographical region, and provides implications for health impacts as well as for current and future risk management related to drought exposure. We used a two-stage statistical model to characterize adverse and protective health impacts for the general population and for subgroups stratified based on age, race, sex, and urbanicity across geographically and climatologically different regions. Our large-scale study of U.S. climate regions emphasizes the poorly studied associations between drought exposure and respiratory mortality in the general population.

Our primary finding is that while drought exposure has a significant impact on the risk of respiratory mortality, the effects vary across climate regions. The results demonstrate spatial heterogeneity, and show that certain regions experience harmful effects from different levels of drought exposure while in other regions show protective or null effects (Figure 2 and Table S1). We presume that the protective or null effects are the result of more complex interactions between drought and mortality in specific locations. More research is required to determine whether or not a potential association exists. One possibility could be that changes in the environment during the drought period might have a protective effect in certain regions. Alternatively, disparities in drought-related mortality could be attributed to differences in adaptive capacity and resilience. Proactive approaches such as early warning systems, efficient healthcare systems, and easily available public health interventions may better equip certain regions to mitigate the negative effects of drought on mortality. Thus, because of their ability to respond successfully to drought-related challenges, in certain locations there may be protective or null effects. In our study, four climate regions (Northeast, Northern Rockies and Plains, Ohio Valley, and Upper Midwest) showed greater risk of respiratory mortality with severe drought, while the Ohio Valley showed decreased risk during severe USDM drought conditions. The other four regions (Northwest, South, Southwest, and West) generally had lower risk from severe droughts. However, the reasons for these regional differences are not entirely clear, and further research is needed to understand these variations. The Northeast and Upper Midwest consistently showed increased health risk with all three drought exposure types.

We found that geographical regions with fewer drought months derived from USDM drought categories had a higher respiratory mortality risk ratio, which is in line with Berman et. al (2017) [16]. However, we did not reach the same conclusion when considering EDDI drought metrics. EDDI captured approximately 15% to 20% more drought events compared to USDM in the Northeast, Ohio Valley, and Upper Midwest, as depicted in Figure 1. Past work has shown that longer drought events are associated with greater relative risk (RR) of respiratory mortality [19]. Although our results support this conclusion, we found that severe 6-month EDDI was associated with relatively higher risk than severe 12-month EDDI (Figure 2 and Table S2).

Our study showed that drought affects populations in different ways. Older populations showed an increased risk of respiratory mortality during high-intensity EDDI droughts (Figure 3 and Table S3). Other studies have demonstrated that the age group over 65 years old is particularly vulnerable to the impacts of drought exposure [16,21,22]. This might be due to a higher baseline mortality rate, pre-existing health conditions (e.g., comorbidity), or higher sensitivity to outdoor conditions. However, individuals between the ages of 40 and 64 showed decreased risk of respiratory mortality during severe USDM and 12-month EDDI droughts. A recent study by Salvador et al. (2021) provided a similar assessment for the 45–64 age group [21]. While a similar conclusion was found in Nebraska [14], the authors applied another layer of stratification based on sex and urbanicity to the 45–54 and 55–64 age groups. We found that respiratory mortality increased for the White populations subgroup during severe EDDI drought and for the Black population subgroup during moderate USDM and severe 6-month EDDI drought (Figure 3 and Table S3). A significantly increased respiratory risk ratio was seen in both the male and female subgroups for severe 6-month and 12-month EDDI and moderate USDM drought (Figure 3 and Table S5). While other studies have demonstrated that increased drought severity has harmful health effects, these studies found that drought exposure significantly affected either male or female populations [22,33,34,35]. We found that both metro and non-metro counties had an increased risk ratio with EDDI drought. However, non-metro counties showed a higher risk ratio and were more susceptible to the impact of drought exposure (Figure 3 and Table S6). Other research has identified similar trends for different health outcomes, including mental health outcomes related to farmers’ job stress [36] and suicide among rural populations [34,37,38,39]. This is potentially due to the direct and indirect effects of drought conditions, the higher reliance on the land for livelihoods in rural regions, and a lack of health care providers and resources.

While there are more than 200 drought indices available for measuring drought status, there is limited research identifying the differences among these drought indicators for health outcome estimation in the public health field [17]. Most of the existing body of research adapts the Standardized Precipitation Index (SPI) and the Standardized Precipitation Evapotranspiration Index (SPEI) for performance assessment [19,20,21,22,36]. However, we used USDM and EDDI in our assessment. The USDM is a robust measure often used in comparison with other drought metrics [27,28]. However, it differs across all NOAA climate regions when compared to the EDDI drought index, as shown in Figure 1. EDDI is a relatively new drought index that has been found to provide earlier warning of drought than the USDM by focusing on the persistence of evaporative demand in a region over three timescales (1, 6, and 12 months) [30]. When using USDM and EDDI to identify associations with respiratory mortality, we found that the direction of these associations differed by NOAA climate region. A previous study found similar result in health risk assessment, although they compared different USDM drought conditions with daily-level health outcomes in the western U.S. [16]. As each drought metric uses unique characteristics to represent the status of drought across regions, there is room for further analysis of health risk assessment with a more complete set of drought metrics that span the hydrological cycle. Our findings suggest that the most suitable indicators may vary by region.

The results of our study have important implications for public health policy and decision-making related to drought exposure [40]. Drought is a complex kind of environmental exposure that can impact human health, human societies, natural resources, and ecosystems both directly and indirectly [15,33]. As previously reported and discussed in this study, the impact of drought on health risk significantly varies across different regions and populations. Therefore, it is important for policymakers, communities, and individuals to develop strategies and guidelines to mitigate the impacts of drought and build resilience to its effects across regions and populations for future management.

We understand that concurrent or compound events throughout the drought period may have a greater influence on the mortality risk ratio. There are a wide range of compound occurrences that can negatively impact human health [5]. Recent studies on the west coast of the United States [41] have shown that heatwaves and drought exacerbate rising concentrations of air pollution and cause health damage, and these concurrent experiences have been shown to result in an increase in mortality and premature births in Brazil [42]. While it is not our primary concern, our sensitivity analysis evaluating using a 90th-percentile temperature-adjusted high-temperature event threshold demonstrated an estimated risk ratio quite similar to the overall risk ratio (see Table S7 in the Supplementary Materials). However, a true assessment of this relationship would be challenging based on the limitations of our data.

This study has several limitations that should be noted. First, our comprehensive assessment was based on an ecological study design, which means that primary analysis was restricted to aggregated data at the county level rather than the individual level. This could potentially result in ecological fallacy. Second, we did not include meta-predictors in the second-stage model due to lack of data availability. Multiple variables, such as climate conditions, socioeconomic factors, and environmental performance index (EPI), can be included simultaneously in the model; however, it is important to be cautious of potential correlations between meta-predictors, and factor analysis might be considered to address this. Moreover, the role of meta-predictors could be examined more carefully to improve our assessment. Third, only two different drought metrics, one a composite measure and one focused on evaporation, were used to assess the risk of respiratory mortality, which may or may not be well-suited for assessments. Further research with more drought indicators is needed.

## 5. Conclusions

Our results indicate that drought can impact respiratory mortality risk based on demographics and climate regions. Our findings suggest that the elderly population, both male and female populations, and White and Black populations are more vulnerable to the effects of drought exposure. Furthermore, residents in non-metro counties have higher risk of respiratory death. In the Northeast and Upper Midwest, all three drought indicators elevate the risk ratio of respiratory fatality. The Northern Rockies and Plains and Ohio Valley have a higher risk ratio for severe drought, whereas the Northwest, South, Southwest, and West have lower risk.

This information could be useful for public health practitioners in providing early warnings and designed messaging to populations of higher concern. We believe that there is more opportunity for future work to better explore and understand these health outcomes. As climate change affects the severity and duration of drought events, it is our hope that this information will be considered for future planning efforts.

## Figures and Tables

**Table 1:** Summary of respiratory death counts for study population during study period (2000–2018). N is the number of respiratory deaths by each subgroup and SD indicates standard deviation.

Race	Age Group	Male			Female		
		*N*	Mean	SD	*N*	Mean	SD
**Total**		2,241,979	9833.24	1685.05	2,420,797	10,617.53	2045.28
White	0–19	9144	40.11	13.83	6954	30.50	12.38
	20–39	15,984	70.11	22.18	13,103	57.47	19.32
	40–64	272,667	1195.91	259.08	239,458	1050.75	249.62
	+65	1,711,596	7507.00	1260.99	1,939,091	8504.79	1639.32
**White Total**		2,009,391	8813.12	1504.73	2,198,606	9643.01	1860.48
Black	0–19	744	3.26	2.25	573	2.51	1.82
	20–39	905	3.97	3.50	646	2.83	1.89
	40–64	7240	31.75	10.85	5560	24.39	8.95
	+65	40,614	178.13	57.32	35,199	154.38	54.86
**Black Total**		49,503	217.12	67.97	41,978	184.11	63.38
Others	0–19	5339	23.42	5.85	3713	16.29	5.09
	20–39	6257	27.44	6.45	5138	22.54	6.96
	40–64	47,807	209.68	42.15	41,976	184.11	45.57
	+65	123,682	502.46	89.39	129,386	567.48	108.28
**Others Total**		183,085	803.00	131.52	180,213	790.41	152.33

**Table 2:** Baseline characteristics for frequency of different drought exposure levels during the study period (2000–2018); n represents the number of counties in each climate region. Note that No, M, and S indicate monthly frequencies of no drought, moderate drought, and severe drought conditions. Abbreviations indicate climate regions (NE: Northeast, UM: Upper Midwest, OV: Ohio Valley, SE: Southeast, NR: Northern Rockies and Plains, S: South, SW: Southwest, NW: Northwest, W: West).

Climate Region	County *n*	USDM			6-Month EDDI			12-Month EDDI		
		No	M	S	No	M	S	No	M	S
Total	3107	521,813	139,047	47,536	465,495	171,999	70,902	443,610	195,772	69,014
NE	245	50,638	4852	370	37,668	11,228	6964	34,557	13,589	7114
		(90.7%)	(8.7%)	(0.7%)	(67.4%)	(20.1%)	(12.5%)	(61.9%)	(24.3%)	(13.8%)
NR	291	43,575	16,598	6176	48,578	13,324	4546	47,271	14,407	4670
		(65.7%)	(25.0%)	(9.3%)	(73.2%)	(19.9%)	(6.9%)	(71.2%)	(21.7%)	(7.0%)
NW	119	17,136	8004	1992	21,405	4566	1161	21,009	4626	1497
		(63.2%)	(29.5%)	(7.3%)	(78.9%)	(16.8%)	(4.3%)	(77.4%)	(17.0%)	(5.5%)
OV	667	131,744	17,725	2607	93,700	40,194	18,182	88,925	46,136	17,015
		(86.6%)	(11.7%)	(1.7%)	(61.6%)	(26.4%)	(12.0%)	(58.5%)	(30.3%)	(11.2%)
S	657	98,560	35,761	15,475	95,239	39,179	15,378	92,430	42,532	14,834
		(65.8%)	(23.9%)	(10.3%)	(63.6%)	(26.2%)	(10.3%)	(61.7%)	(28.4%)	(9.9%)
SE	572	93,311	26,616	10,489	83,306	33,082	14,028	76,939	39,951	13,526
		(71.5%)	(20.4%)	(8.0%)	(63.9%)	(25.4%)	(10.8%)	(59.0%)	(30.6%)	(10.4%)
SW	140	15,285	10,864	5771	22,639	6417	2864	22,510	6780	2630
		(47.9%)	(34.0%)	(18.1%)	(70.9%)	(20.1%)	(9.0%)	(70.5%)	(21.2%)	(8.2%)
UM	341	63,456	12,832	1460	51,630	19,735	6383	49,264	22,760	5724
		(81.6%)	(16.5%)	(1.9%)	(66.4%)	(25.4%)	(8.2%)	(63.4%)	(29.3%)	(7.4%)
W	75	8108	5796	3196	11,330	4374	1396	10,705	4991	1404
		(47.4%)	(33.9%)	(18.7%)	(66.3%)	(25.6%)	(8.2%)	(62.6%)	(29.2%)	(8.2%)

## Data Availability

The mortality data can be requested from the Center for Disease Control and Prevention (CDC) at the following (accessed on 3 January 2023): https://www.cdc.gov/nchs/nvss/nvss-restricted-data.htm. There are detailed instructions on how to access this data, including the project review form and supporting materials that need to be submitted. In order to access the health data, all members must sign a Data Use Agreement (DUA).

## Supplementary Materials

The following supporting information can be downloaded at: https://www.mdpi.com/article/10.3390/ijerph20126076/s1, Figure S1: The percentage of experiencing drought events by different drought exposures in the U.S. (2000-2018); Table S1: Overall effects of different drought exposures to respiratory mortality outcomes by NOAA climate regions. The top value is risk ratio and the value in parentheses indicates the credible interval of the estimated risk ratio; Table S2: The number counties used in the second stage model (final analysis); Table S3: Overall effects of different drought exposures to health effects by age group; Table S4: Overall effects of different drought exposures to health effects by race; Table S5: Overall effects of different drought exposures to health effects by sex; Table S6: Overall effects of different drought exposures to health effects by urbanicity; Table S7: Sensitivity analysis for overall effects of different drought exposures to respiratory mortality outcomes by NOAA climate regions. The top value is risk ratio and the value in parentheses indicates the credible interval of the estimated risk ratio.

## Abbreviations

The following abbreviations are used in this manuscript:

NOAA	National Oceanographic and Atmospheric Administration
USDM	United States Drought Monitor
EDDI	Evaporative Demand Drought Index
Cr	Credible Interval
NE	Northeast
UM	Upper Midwest
OV	Ohio Valley
SE	Southeast
NR	Northern Rockies and Plains
S	South
SW	Southwest
NW	Northwest
W	West
