# Supplementary material for: The Association between Drought Exposure and Respiratory-Related Mortality in the United States from 2000 to 2018
Source: Int J Environ Res Public Health. 2023 Jun 7;20(12):6076. doi: 10.3390/ijerph20126076 (PMC10298035; doi:10.3390/ijerph20126076)
Supplement: Supplementary file 1 [file ijerph-20-06076-s001.zip › ijerph-2371056-supplementary.pdf]

Supplementary Materials

This supplementary material provides additional information.

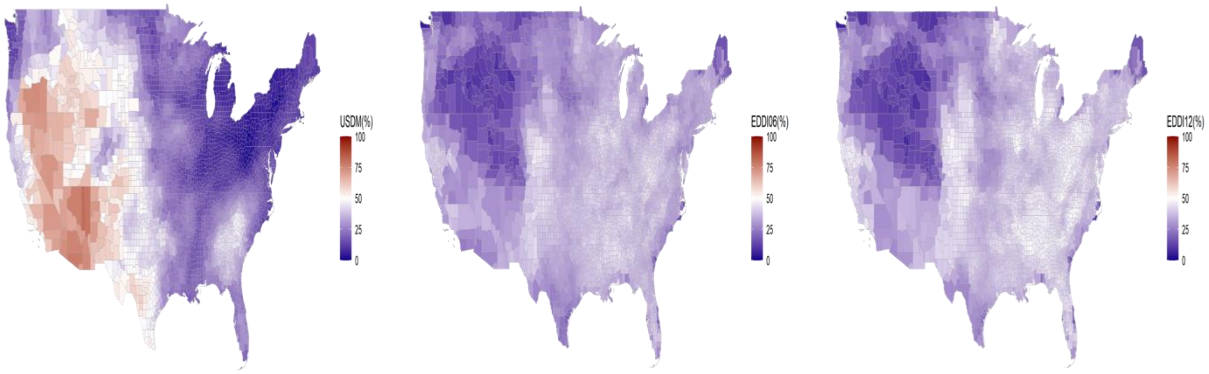

Figure S1. The percentage of experiencing drought events by different drought exposures in the U.S. (2000-2018).

Table S1. Overall effects of different drought exposures to respiratory mortality outcomes by NOAA climate regions. The top value is risk ratio and the value in parentheses indicates the credible interval of the estimated risk ratio. Note that the values are log-scale and need to take exponentiate as described in Method Section.

Climate Region	USDM		6-month EDDI		12-month EDDI	
	**Moderate**	**Severe**	**Moderate**	**Severe**	**Moderate**	**Severe**
NE	0.011<br>(-0.001,0.022)	0.023<br>(-0.012,0.059)	0.013<br>(0.004,0.021)	0.060<br>(0.048,0.072)	0.015<br>(0.006,0.023)	0.036<br>(0.022,0.048)
NR	0.002<br>(-0.024,0.027)	0.090<br>(0.049,0.133)	0.007<br>(-0.022,0.034)	0.073<br>(0.021,0.125)	-0.037<br>(-0.069,-0.009)	0.067<br>(0.014,0.114)
NW	-0.088<br>(-0.111,-0.064)	-0.104<br>(-0.163,-0.044)	0.001<br>(-0.02,0.023)	0.006<br>(-0.036,0.047)	0.001<br>(-0.022,0.023)	-0.064<br>(-0.106,-0.023)
OV	0.015<br>(0.004,0.025)	-0.037<br>(-0.064,-0.008)	-0.008<br>(-0.016,0.000)	0.041<br>(0.028,0.053)	0.009<br>(0.000,0.018)	0.052<br>(0.039,0.065)
S	0.014<br>(0.004,0.024)	-0.009<br>(-0.029,0.01)	-0.035<br>(-0.048,-0.025)	-0.032<br>(-0.050,-0.013)	-0.025<br>(-0.039,-0.012)	-0.046<br>(-0.064,-0.026)
SE	0.018<br>(0.008,0.026)	0.005<br>(-0.009,0.020)	0.014<br>(0.006,0.023)	0.035<br>(0.019,0.050)	0.021<br>(0.013,0.029)	-0.001<br>(-0.017,0.016)
SW	0.013<br>(-0.009,0.038)	-0.103<br>(-0.135,-0.069)	-0.018<br>(-0.038,0.002)	0.021<br>(-0.014,0.053)	-0.061<br>(-0.085,-0.040)	-0.076<br>(-0.111,-0.041)
UM	0.012<br>(-0.003,0.026)	0.159<br>(0.108,0.204)	0.028<br>(0.017,0.041)	0.095<br>(0.075,0.115)	0.009<br>(-0.005,0.022)	0.073<br>(0.050,0.098)
W	-0.095<br>(-0.124,-0.068)	-0.074<br>(-0.118,-0.028)	-0.021<br>(-0.037,-0.005)	-0.004<br>(-0.034,0.026)	-0.060<br>(-0.078,-0.043)	-0.071<br>(-0.104,-0.039)

For statistical analysis, we applied strict threshold based on population size in counties that are greater than 25,000 and 100,000. The approach provided 1,635 and 597 counties to be included in the first stage modeling. However, we need to remove more counties after the first stage model due to convergence issue, which typically occurred in modeling count data. As discussed in the main article, we used a different criterion to include more counties in the analysis. Table S2 shows the number of counties to be included in the final analysis, more specially, in the second stage Bayesian meta-analysis. Our proposed approach maintained much more counties compared with strict threshold technique.

Table S2. The number counties used in the second stage model (final analysis).

Climate Region	USDM		6-month EDDI		12-month EDDI	
	Moderate	Severe	Moderate	Severe	Moderate	Severe
Total Counties (n=3,107)	3,034	2,824	3,044	2,926	3,037	2,886
NE	243	242	244	244	245	243
NR	265	232	264	213	268	212
NW	114	108	115	100	112	105
OV	662	581	665	660	667	661
S	643	620	642	618	635	598
SE	565	537	570	563	569	555
SW	130	122	130	124	128	118
UM	340	315	340	332	339	326
W	72	67	74	72	74	68

Table S3. Overall effects of different drought exposures to health effects by age group.

Age Group	USDM		6-month EDDI		12-month EDDI	
	Moderate	Severe	Moderate	Severe	Moderate	Severe
0-19	-0.011 (-0.024,0.001)	-0.004 (-0.028,0.020)	-0.008 (-0.019,0.005)	-0.004 (-0.023,0.016)	-0.006 (-0.019,0.007)	-0.001 (-0.022,0.022)
20-39	-0.003 (-0.016,0.012)	-0.001 (-0.027,0.026)	-0.007 (-0.02,0.006)	-0.004 (-0.027,0.019)	-0.009 (-0.023,0.006)	-0.015 (-0.038,0.011)
40-64	0.005 (-0.004,0.015)	-0.024 (-0.043,-0.005)	-0.011 (-0.019,-0.002)	0.013 (0.000,0.026)	-0.006 (-0.015,0.003)	-0.021 (-0.036,-0.006)
+65	0.007 (0.002,0.012)	-0.002 (-0.012,0.009)	0.003 (-0.001,0.007)	0.038 (0.032,0.045)	0.001 (-0.003,0.006)	0.020 (0.013,0.027)

Table S4. Overall effects of different drought exposures to health effects by race.

Race	USDM		6-month EDDI		12-month EDDI	
	Moderate	Severe	Moderate	Severe	Moderate	Severe
White	0.005 (0.000,0.01)	-0.005 (-0.015,0.006)	0.001 (-0.003,0.004)	0.035 (0.029,0.042)	0.001 (-0.003,0.005)	0.014 (0.007,0.021)
Other	-0.004	-0.003	-0.014	0.001	-0.013	-0.005

	(-0.016,0.007)	(-0.027,0.018)	(-0.025,-0.003)	(-0.017,0.018)	(-0.025,-0.001)	(-0.024,0.014)
Black	0.010<br>(0.001,0.020)	0<br>(-0.019,0.019)	0.007<br>(-0.002,0.016)	0.032<br>(0.017,0.046)	0<br>(-0.009,0.010)	0.003<br>(-0.013,0.018)

Table S5. Overall effects of different drought exposures to health effects by sex.

Gender	USDM		6-month EDDI		12-month EDDI	
	Moderate	Severe	Moderate	Severe	Moderate	Severe
Male	0.008<br>(0.001,0.014)	-0.011<br>(-0.023,0.000)	-0.002<br>(-0.007,0.003)	0.028<br>(0.020,0.036)	-0.001<br>(-0.006,0.004)	0.008<br>(0.000,0.016)
Female	0.006<br>(0.000,0.012)	-0.005<br>(-0.017,0.007)	0.003<br>(-0.002,0.008)	0.042<br>(0.034,0.050)	0.001<br>(-0.004,0.007)	0.017<br>(0.008,0.025)

Table S6. Overall effects of different drought exposures to health effects by urbanicity.

Urbanicity	USDM		6-month EDDI		12-month EDDI	
	Moderate	Severe	Moderate	Severe	Moderate	Severe
Metro	0.003<br>(-0.002,0.009)	-0.018<br>(-0.029,-0.006)	-0.001<br>(-0.005,0.004)	0.034<br>(0.026,0.041)	-0.001<br>(-0.006,0.004)	0.009<br>(0.001,0.016)
Non-metro	0.012<br>(0.004,0.021)	0.021<br>(0.004,0.037)	0.007<br>(-0.001,0.015)	0.043<br>(0.031,0.055)	0.010<br>(0.002,0.018)	0.032<br>(0.020,0.045)

Table S7. Result for sensitivity analysis: Overall effects of different drought exposures to respiratory mortality outcomes by NOAA climate regions. The top value is risk ratio and the value in parentheses indicates the credible interval of the estimated risk ratio. Note that the values are log-scale and need to take exponentiate as described in Method Section.

Climate Region	USDM		6-month EDDI		12-month EDDI	
	Moderate	Severe	Moderate	Severe	Moderate	Severe
NE	0.010<br>(-0.001,0.022)	0.032<br>(-0.002,0.067)	0.014<br>(0.005,0.022)	0.062<br>(0.049,0.073)	0.015<br>(0.006,0.024)	0.037<br>(0.025,0.050)
NR	0.004<br>(-0.021,0.029)	0.093<br>(0.051,0.138)	0.007<br>(-0.021,0.033)	0.073<br>(0.022,0.126)	-0.036<br>(-0.066,-0.006)	0.069<br>(0.021,0.118)
NW	-0.089<br>(-0.111,-0.067)	-0.103<br>(-0.160,-0.040)	0.000<br>(-0.022,0.021)	0.002<br>(-0.040,0.046)	0.001<br>(-0.021,0.025)	-0.065<br>(-0.106,-0.022)
OV	0.015<br>(0.004,0.026)	-0.037<br>(-0.067,-0.011)	-0.007<br>(-0.016,0.001)	0.040<br>(0.028,0.052)	0.009<br>(0.000,0.018)	0.051<br>(0.038,0.065)
S	0.014<br>(0.003,0.024)	-0.010<br>(-0.031,0.009)	-0.034<br>(-0.046,-0.022)	-0.030<br>(-0.049,-0.013)	-0.025<br>(-0.039,-0.011)	-0.044<br>(-0.063,-0.025)
SE	0.018<br>(0.008,0.027)	0.005<br>(-0.009,0.020)	0.014<br>(0.006,0.022)	0.035<br>(0.018,0.050)	0.020<br>(0.012,0.028)	-0.001<br>(-0.017,0.015)
SW	0.014<br>(-0.009,0.036)	-0.103<br>(-0.140,-0.070)	-0.018<br>(-0.040,0.000)	0.020<br>(-0.013,0.053)	-0.062<br>(-0.083,-0.041)	-0.076<br>(-0.109,-0.039)
UM	0.012<br>(-0.003,0.027)	0.157<br>(0.110,0.204)	0.029<br>(0.017,0.041)	0.098<br>(0.075,0.120)	0.010<br>(-0.003,0.024)	0.074<br>(0.050,0.099)

W	-0.094<br>(-0.122,-0.067)	-0.079<br>(-0.121,-0.035)	-0.023<br>(-0.039,-0.007)	0.002<br>(-0.028,0.032)	-0.057<br>(-0.075,-0.040)	-0.070<br>(-0.101,-0.038)
